# The role of social acuity assessment in differentiating primary psychoses from drug-induced psychoses

**DOI:** 10.1192/j.eurpsy.2021.645

**Published:** 2021-08-13

**Authors:** V. Ciobanu, M. Minciună, B. Bucatos, M. Ciotu, C. Sudrijan, A. Olteanu, A.-M. Bortun, M. Bondrescu, I. Papavă, A.-M. Romosan, R. Romosan, L. Dehelean

**Affiliations:** 1 Psychiatry, Victor Babes University of Medicine and Pharmacy Timisoara, Timisoara, Romania; 2 Psychiatry, “Pius Brânzeu” Emergency County Hospital, Timisoara, Romania; 3 Psychiatry Ii, Pius Brinzeu Emergency County Hospital, Timisoara, Romania

**Keywords:** dual diangosis, substance-induced psychosis, schizophrénia, bipolar disorder

## Abstract

**Introduction:**

The dual diagnosis among patients with primary psychotic disorders is frequent and causes diagnostic and treatment challenges. In clinical practice, differentiating between substance-induced psychoses and independent (primary) psychoses when the patient is actively using drugs of addiction, is difficult, especially in the acute phase of the psychosis.

**Objectives:**

The aim of the study is to identify clinical data relevant for differentiating between primary psychoses triggered by addictive drug misuse and substance-induced psychoses, using psychometric scales.

**Methods:**

The study was conducted on 111 patients divided in four samples: 28 dual diagnosis psychotic patients (DD), 27 bipolar patients (BD), 25 schizoaffective patients (SCA) and 31 patients with schizophrenia (SCZ). The subjects were assessed using scales for the severity of psychiatric symptoms, cognitive functions and social acuity (theory of mind): BPRS-E (Brief Psychiatric Rating Scale – Expanded), MoCA (Montreal Cognitive Assessment), CBS (Cambridge Behavioral Scale), and RMET (Reading the Mind in the Eyes Test). The tests were performed when patients were in the remission phase of the psychosis.

**Results:**

BPRS-E scores showed significant differences between DD subjects and patients from the other three samples (primary psychoses). CBS revealed significant differences between the DD subjects and patients with schizophrenia spectrum psychoses (SCA and SCZ). RMET identified significant differences between DD and BD patients.

**Conclusions:**

Although differentiating between substance-induced and primary psychoses remains a difficult task, social acuity assessment performed in remitted patients may be helpful in guiding the clinician to establish a more accurate diagnosis.

